# MRI-Based Medical Image Recognition: Identification and Diagnosis of LDH

**DOI:** 10.1155/2022/5207178

**Published:** 2022-09-09

**Authors:** Shuai Wang, Zhengwei Jiang, Hualin Yang, Xiangrong Li, Zhicheng Yang

**Affiliations:** ^1^College of Mechanical and Electrical Engineering, Qingdao University of Science and Technology, Qingdao 266061, China; ^2^Department of Radiology, Qilu Hospital (Qingdao), Cheeloo College of Medicine, Shandong University, Qingdao, China

## Abstract

To realize the automatic symptom recognition and classification of MR images and improve the accuracy and efficiency of the diagnosis of lumbar intervertebral disc herniation (LDH), a method for lumbar intervertebral disc recognition and disease classification is proposed in this paper. The method mainly includes three steps: preprocessing, target segmentation, and symptom classification. Preprocessing is performed by noise reduction and interference removal methods for blurred images. The contour poles are used to determine the four points of the tail vertebra in order to reduce the wrong segmentation of the tail vertebra. A classification method based on five judgment indicators is proposed, which effectively improves the stability of disease diagnosis. The example verifies that the algorithm can accurately complete the target segmentation and the accuracy of symptom classification reaches the standard of professional doctors, which proves that the method has good robustness.

## 1. Introduction

Lumbar disc herniation (LDH) is a very common disease of the lumbar spine, with the main causes being degenerative changes and injuries of the lumbar disc [1]. The diagnosis process of lumbar disc herniation include initial diagnosis and confirmation, and the whole process is cumbersome and time-consuming, and it is difficult to guarantee the real-time application and accuracy for diagnosis with the huge number of patients and the uneven levels of doctors. The efficiency and accuracy of the initial diagnosis have become the bottleneck problem in the diagnosis of LDH. Therefore, the algorithm proposed in this paper for lumbar disc recognition and disease classification could be achieved by three steps including preprocessing, target segmentation and recognition, and symptom classification. With this method, the accurate identification, classification, and diagnosis for LDH are realized to assist doctors.

Target segmentation and recognition is a key step in processing patient image data analysis, which helps in the next step of symptom diagnosis and treatment plan [2–4]. Classical medical image segmentation techniques can be classified as threshold-based segmentation [5], edge or boundary-based segmentation [6], region-based segmentation [7, 8], active contour model-based techniques [9, 10], and neural network-based segmentation [11–15]. To increase the standardization and normality of the diagnosis, the severity of the disease is classified by some scholars. Current classification systems are based on imaging and pathomorphism [16–21], and LDH is classified as bulge, protrusion, and extrusion according to the degree of prominence of the injury. In addition, there are nonruptured, ruptured, and sequestered types based on surgical pathomorphism. Wiltse et al. described the size of the lesion which can be assessed by normal, mild, moderate, moderately severe, and severe based on the size and location of lesions in the lumbar or thoracic spine [22]. Milette studied the imaging and pathological presentation of lumbar disc herniation and the size of the disc and the location of the herniation to standardize the nomenclature of their types [23]. Mysliwiec et al. of Michigan State University proposed a simple, objective classification method that expresses the location distribution of herniated discs longitudinally and laterally, respectively, taking into account both the size of the herniated disc and its location in local anatomical conditions [24]. Kaliya-Perumal et al. used the Michigan State University (MSU) lumbar disc herniation (LDH) classification to classify lumbar disc herniations and determined the reliability of this classification system among orthopedic residents at the institute [25]. In 2020, Gupta et al. determined that the cross-sectional area provided a more reliable measurement modality for DiskLDHS compared to linear measurements of anterior and posterior lengths, so the cross-sectional area and its characterization of LDH is superior in its characterization [26]. On the other hand, Hao et al. established new grading and classification criteria for LDH, which combined the patient's clinical manifestations and imageology for LDH which can enable accurate assessment [17]. Divi et al. classified the morphology of disc herniation according to the type, size, and location of the herniated annoyance to determine the predictive factors for surgical intervention of LDH [27]. However, several of the above methods still lack the mode of automatic image recognition followed by automatic classification and diagnosis to give the initial diagnosis and lack the management process from image recognition to classification and diagnosis, which leads to the design of algorithms for each part without considering the application of other parts of the process and thus cannot be effectively applied in practice. In this paper, the features of vertebrae and intervertebral discs including contours based on the improved segmentation and recognition algorithms are extracted, and we also propose five symptom recognition indicators to be applied to the LDH images after automatic recognition and obtain an effective algorithm that can generate the initial diagnosis report. The method optimizes the combination between the steps and achieves better results even for blurred images.

**Figure 1:**
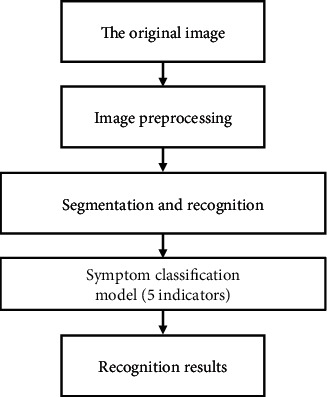
Flow chart of LDH diagnosis.

T2-weighted images are used in this paper, and the algorithm model is written and implemented by Python3.0 combined with OpenCV library. The whole diagnostic process is shown in Figure 1.

## 2. Image Preprocessing

MRI images contain vertebrae, intervertebral discs, spinal canal, muscles, nerves, and other tissues, and accurate recognition for vertebrae and intervertebral discs is the first step for successful diagnosis. Therefore, preprocessing of images to remove noise and interference is essential for better quality segmentation of vertebrae and discs afterwards.

**Figure 2:**
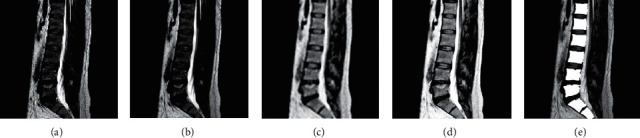
(a) Original image. (b) Image after open operation. (c) Image after gamma transformation. (d) Effect of blurring transformation. (e) Effect of histogram equalization.

(1) The gray-level open operation was performed on some original images shown in Figure 1(a) on account of the bright noise existing in the right side of the vertebrae. Elliptical structures with a size of 3 × 3 were selected, and the effect that the noise obviously darkened after filtering is shown in Figure 2(b).
(2) Gamma transform was performed for overall brightness improvement in order to solve the difficulty of the separation between the low grayscale values target and low-light level of the background of the MRI images. The basic formula of gamma transform is
  (1) $$\begin{array}{c} s=cr^{\gamma }\text{.} \end{array}$$
In the formula, s is the output of gray level, *r* is the input of gray level, and the offset *c* is set to zero.
For Figure 2(b), the gamma transformation was performed by choosing *γ* = 0.5 and *c* = 1, and the results are shown in Figure 2(c).
(3) Fuzzy transformation of Figure 2(c), whose results are shown in Figure 2(d), shows the gray value near the middle is separated, increasing the contrast of the image; in some areas, there is an over “exposure” phenomenon, but it did not affect the subsequent binarization process.
(4) Finally, the equalizeHist function in the openCV library is used for histogram equalization, and Figure 2(e) shows the processing results.

## 3. Segmentation and Recognition of the Vertebrae

Since relative location relationship between the intervertebral discs and vertebrae was the key for LDH diagnosis, the boundary information of each intervertebral disc and vertebra should be extracted separately with the segmentation and recognition of intervertebral discs and vertebrae separately.

### 3.1. Initial Treatment of Vertebrae

In this paper, the multithresholding method and a grayscale threshold binarization method is used to achieve the segmentation of vertebrae [28, 29].

#### 3.1.1. Initial Treatment of Vertebrae

**Figure 3:**
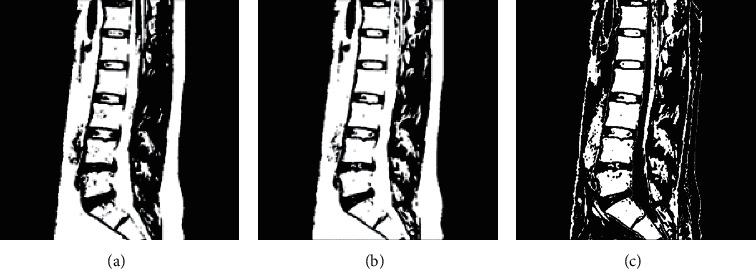
(a) Basic global threshold processing. (b) Otsu method. (c) Multithreshold processing.

For blurred images, the contrast of the image is enhanced through the preliminary processing of grayscale image binarization. The common methods of grayscale threshold binarization include basic global thresholding, the Otsu method, and multithresholding, and the effect is shown in Figure 3 by applying the above three methods to Figure 2(e).

**Figure 4:**
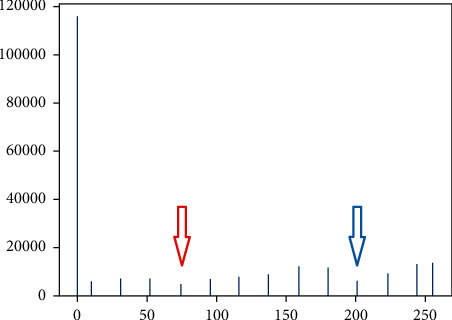
Gray distribution.

As the result, the segmentation effect of the multithresholding process was better than others. The process of multithresholding is as follows: first, the grayscale histogram of the image was drawn after image preprocessing, and there were two troughs shown by the red and blue arrows in Figure 4 near the grayscale values of 80 and 200. The grayscale value of the second trough was exactly the threshold required for target binarization, while another threshold is obtained by a large number of experiments around 105. For example, the best segmentation results are achieved when 105 and 205 are chosen as thresholds for the MRI of Figure 2(a).

**Figure 5:**
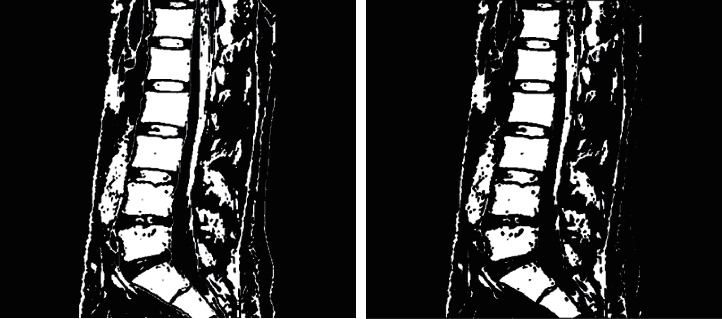
The effect of corrosion operation.

In order to avoid the recognition error caused by the vertebral adhesions, corrosion was used for disconnecting the adhesion region and the same size structural element for the expansion. The interference of adhesions between vertebrae was effectively removed as Figure 5 before and after corrosion.

#### 3.1.2. Screening of Vertebral Contours

The vertebral contours fixed and containing some unique features such as area features, shape features, and so on were screened based on the features. Through statistical analysis of 356 samples obtained from the hospital, the approximate parameter range of the vertebral contour is determined, which is used as a condition to screen the contour.

**Figure 6:**
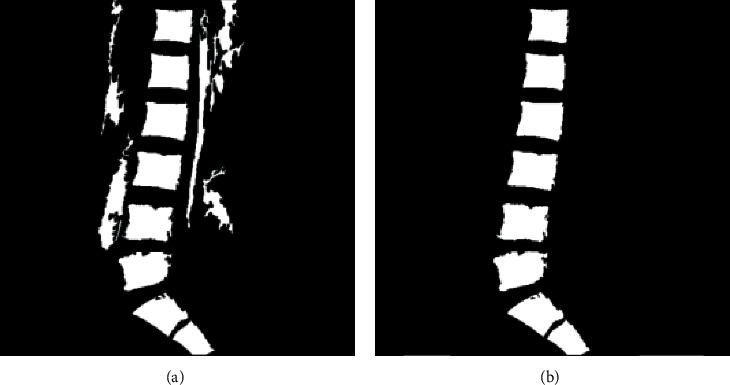
(a) Small area removal method. (b) Screening results.

1. *Screening of Contours Based on Area Features.*
2. The target contours from the binary image were screened based on the contour area limited from 3000 pixels to 6000 pixels. Then most of the contours such as more than 6000 pixels and less than 3000 pixels were eliminated, and the screening results are shown in Figure 6(a).
3. *Screening of Contours Based on Shape Features.*
4. The shape feature was more efficient for nontarget contours eliminated with similar size when compared with that of the area feature. As shown in the Figure 6(a), the vertebrae as the target area were similar to a rectangle, while the shape of the nontarget contours was irregular. Through the statistical analysis, the condition with shape features was determined when contour rectangle aspect ratio was set between 0.6 and 1.4.
5. *Contour Length Was Set Less Than 400.*
6. As an effect after screening, as shown in Figure 6(b), nontarget contours were separated out and eliminated.

#### 3.1.3. Positioning of the Vertebrae

The vertebrae recognized completely need to be positioned as the boundary for determining whether the disc is herniated or not, and since the caudal vertebrae with heeling condition are different from the common lumbar vertebrae, different positioning methods were required.

**Figure 7:**
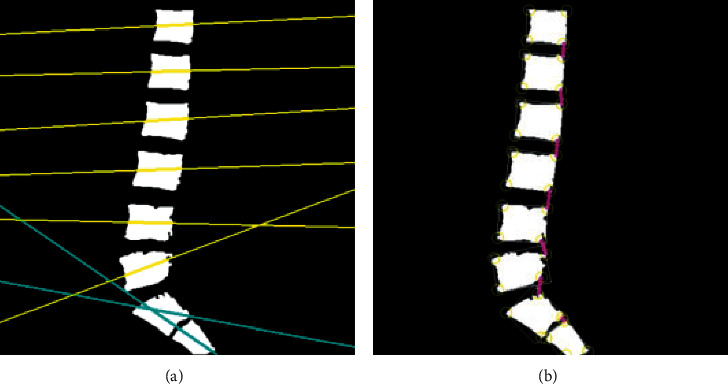
(a) Optimal fitting line. (b) Optimal fitting line.

The characteristic that the ordinary vertebrae were positioned positively while the caudal vertebrae showed an overall inclination, and the best-fitting straight line of the contour is found, and the slope of the straight line from 0.1 to 1 is determined as the range for determining the caudal vertebrae. All the pixel points of each vertebra were acquired with OpenCV, and the coordinate point farthest from the four corners of the picture was found as the target corner point. The caudal vertebrae are tilted in MRI images, so the contour poles were used to determine four points, i.e., the highest, lowest, rightmost, and leftmost points as contour angle points. Each straight line through the vertebrae in Figure 7(a) is the best-fitting straight line, where the blue line is the tail vertebrae fitting straight line.

The results after localization of all vertebrae are shown in Figure 7(b). The locations marked by yellow circles are contour corner points (yellow rectangular boxes are rectangular enclosing boxes), and the pink line segments are the normal demarcation lines of the intervertebral discs.

### 3.2. Segmentation and Recognition of Intervertebral Discs

Compared to vertebrae, intervertebral discs were segmented better with global thresholding and the separation of the discs from the background which was achieved when pixel points with gray values above 50 are transformed to black by 356 images counted.

**Figure 8:**
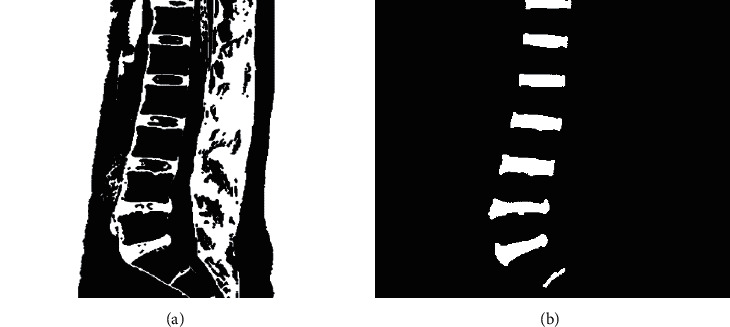
(a) Morphological manipulation. (b) Disc contour.

First, the best segmentation results shown in Figure 8(a) were achieved with elliptical structure elements of size 2 × 2. Then, some nontarget contours, whose area was less than 100 and more than 2000, were removed by the small area removal method. Second, the incidental spine and interspinous ligament on the right side were removed with the rectangular aspect ratio greater than 0.8. Finally, the open operation was performed. The screening effect is shown in Figure 8(b).

## 4. Initial Diagnosis of Disc Herniation

Preliminary diagnosis can be made after the vertebrae and discs are positioned separately. Following indicators were used for the diagnosis.

### 4.1. Diagnosis with Protrusion Distance

**Figure 9:**
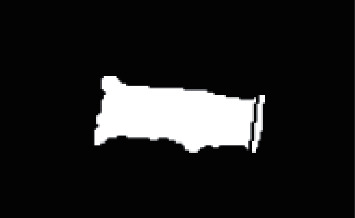
A profile of a disc herniation.

With the demarcation line acquired in Section 3.1.3, each recognized disc herniation contour was divided into protrusion part and normal part. As shown in Figure 9, the area on the right was the protrusion part. If the protrusion part is absent or tiny, protrusion had not yet appeared; otherwise, the farthest distance from the point in the area of disc herniation part to the demarcation line was the protrusion distance of this disc for judging the type of disease.

### 4.2. Diagnosis with the Average Gray Scale

**Figure 10:**
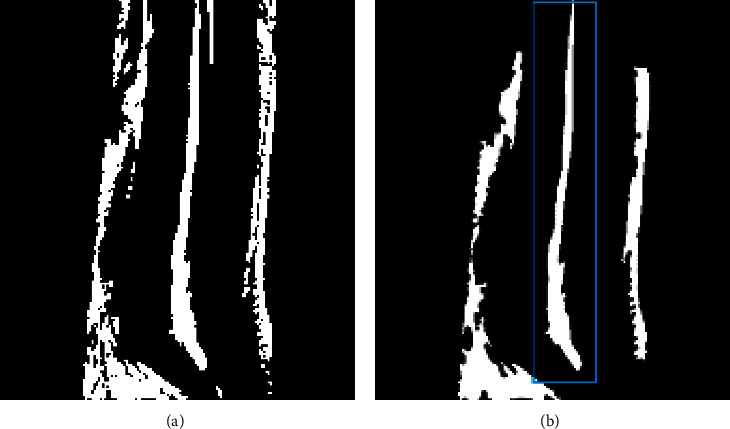
Spinal canal identification. (a) Binarized image. (b) Disc contour.

If the area of a disc in MRI was low grayscale, severe degeneration appeared to diagnose the herniation for this disc. For example, 20 was selected as the threshold value of herniation and the average grayscale calculated for each disc in Figure 10(b) is as follows:

[27.06, 50.83, 55.44, 49.95, 47.41, 24.02, 19.42, 24.56].

The results indicate that the severe degeneration appeared on the seventh intervertebral disc with a mean gray level of 19.42.

### 4.3. Diagnosis with Spinal Canal Recognization

In some cases of extrusion type, the image of spinal canal was disrupted in MRI because of the interruption from the material within the disc moving into the spinal canal. So the presence or absence of disruption in the image of spinal canal acted as an indicator for herniation disc recognition.

The image with binarization was shown as Figure 10(a). Since the vertebrae were close to the spinal canal, an appropriate range was set according to the average *X* coordinate of the far right of the vertebrae. The spinal canal was identified if the *X* coordinate of the far left of each contour was within the setting range. The contour in the blue box in Figure 10(b) was the identified spinal canal which is same as the real spinal canal in shape and size.

## 5. Experimental Results

A preliminary diagnosis can be made by the identification and localization of vertebrae and discs, but this diagnosis is not uniform criteria and not suitable for automatic computerized diagnosis. Therefore, the disease conditions should be classified and the criteria for discriminating each category should be stipulated.

### 5.1. Indicator Selection

Due to a single index such as protrusion distance, as a basis for judgment can easily lead to misclassification, multiple contour features are considered comprehensively and multiple indexes are used to achieve typing of intervertebral disc disease. In this paper, five judgment indexes based on two-dimensional images were proposed with previous research results including protrusion distance, protrusion area, protrusion length ratio, protrusion area ratio, and average grayness.

**Figure 11:**
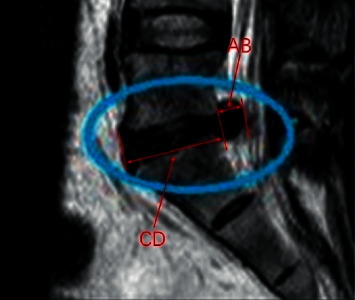
Classification indicators.

1. Protrusion distance: The farthest distance from the protrusion part to the dividing line AB, as shown in Figure 11
2. Protrusion area: Protrusion area of part of the outline
3. Protrusion length ratio: The ratio of the protrusion distance to the length of the entire intervertebral disc in the *X*-axis direction (CD)
4. Protrusion area ratio: The ratio of the protrusion part to the whole disc area
5. Average gray level: Average gray level of the intervertebral disc.

Forty MR images of different patients were selected, and each intervertebral disc in the image was a herniated lesion and a type of herniated lesion to form a labeled MRI. As shown in Table 1, the five indicators of MRI are quantitatively measured, and each indicator accounts for 0.25 of the proportion of symptom assessment. The results show that the five indicators are positively correlated with the three types of protrusion, and each outstanding type of diagnosis satisfies the condition of at least the size of the range containing any three indicators. If in any four of the five indicators, the size of each of the two indicators is within the range of the two types of protrusions;that is, one type of protrusion includes only one indicator, and the other two types of protrusions each include conditions for two different indicators, and it is impossible to determine which type they belong to, and then in accordance with the order of protrusion distance, protrusion area, protrusion length ratio, protrusion area ratio, and average gray scale, the protrusion type corresponding to the top-ranked indicator is the final diagnosed protrusion symptom. According to the above-mentioned conditions, 5 index data are used for disease classification.

### 5.2. Method Validation and Analysis

#### 5.2.1. Overall Algorithm Validation

**Figure 12:**
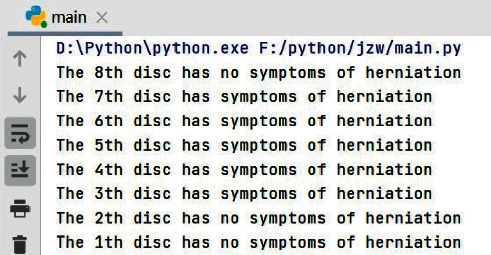
The result of running the program.

The final operation result of the automatic diagnosis is shown in Figure 12. Compared with the results of manual diagnosis, the 7th disc has low brightness and serious degeneration is occurred. The 3rd, 4th, 5th, 6th, and 7th discs in the figure have different degrees of protrusion, and the spinal canal in the images remained continuous and no serious lumbar disc herniation occurred, and the type of herniation of each disc was basically correct, which proved that the algorithm could accomplish the research objectives better. The running result is combined with the numerical value of the degree of lumbar disc herniation and showed whether the spinal canal is continuous as the final report result.

#### 5.2.2. Verify the Accuracy of Target Segmentation

Twenty MR images were randomly selected as the initial data set, and those were preprocessed, and then segmented and identified to obtain the vertebral bone and disc contours, and the statistical results were as follows.

There were a total of 169 vertebrae in the 20 images (removing the top incomplete vertebrae). The algorithm could accurately identify 163 vertebrae, with a recognition success rate of 96.45%. There were a total of 153 intervertebral discs (not counting the tail bones) in the 20 images, and 149 discs were identified, with a recognition success rate of 97.38%. It proves that the image processing algorithm in this paper has certain reliability.

#### 5.2.3. Validation of Five Indicators

**Figure 13:**
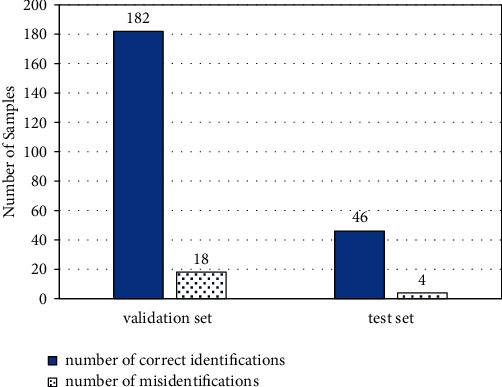
Classification result.

200 images in the validation set and 50 images in the test set were used to verify the accuracy of the model. As shown in Figure 13, in the validation set, the number of correct detections is 182, the number of false detections is 18, and the classification accuracy rate is 91%; in the verification set, the number of correct detections is 46, the number of false detections is 4, and the classification accuracy is 92%, which proves that the classification model can meet the requirements of this research and realizes the classification and diagnosis of diseases.

## 6. Conclusion

In this paper, a new method for LDH automatic diagnosis was proposed to realize the full automation of MR images recognition and diagnosis and provides conclusion of initial diagnosis and forms the basis of final diagnosis for patients and doctors. This method also has a better recognition effect on fuzzy images that are difficult to judge. In addition, five indicators proposed are strongly available for different LDH auxiliary diagnosis. The experimental results show that the classification accuracy of the validation set and test set reached 91% and 92%, respectively, which proves the effectiveness of the method and can provide auxiliary diagnosis for doctors. In the future, we will add other indicators to be used in symptom classification and automatically eliminate unclear MR images to reduce the workload of medical workers.

## Figures and Tables

**Table 1:** Mark the prominent type of indicator data statistics.

	Highlight distance (mm)	Highlighted area (mm^2^)	Prominent length ratio	Prominent area ratio	Average gray level
Bulge	1∼5	10∼100	0.01∼0.1	0.01∼0.1	>35
Protrusion	5∼10	100∼200	0.1∼0.2	0.1∼0.2	15∼35
Extrusion	>10	>200	>0.2	>0.2	<15

## Data Availability

The data that support the findings of this study are available upon request from the corresponding author.
